# Correlates of Persistent Post-Concussive Symptoms: Results From a Nationally Representative Cohort of Canadian Armed Forces Personnel

**DOI:** 10.1093/milmed/usag078

**Published:** 2026-02-28

**Authors:** Meredith J Seager, Tracie O Afifi, Renée El-Gabalawy, Pamela Holens, Jitender Sareen, Shay-Lee Bolton

**Affiliations:** Department of Psychiatry, Max Rady College of Medicine, Rady Faculty of Health Sciences, University of Manitoba, Winnipeg, MB R3E 3N4, Canada; Department of Psychology, Faculty of Arts, University of Manitoba, Winnipeg, MB R3T 2N2, Canada; Department of Psychiatry, Max Rady College of Medicine, Rady Faculty of Health Sciences, University of Manitoba, Winnipeg, MB R3E 3N4, Canada; Department of Community Health Sciences, Max Rady College of Medicine, Rady Faculty of Health Sciences, University of Manitoba, Winnipeg, MB R3E 0T4, Canada; Department of Clinical Health Psychology, Max Rady College of Medicine, Rady Faculty of Health Sciences, University of Manitoba, Winnipeg, MB R3E 3N4, Canada; Department of Anesthesiology, Perioperative and Pain Medicine, Max Rady College of Medicine, Rady Faculty of Health Sciences, University of Manitoba, Winnipeg, MB R3E 0Z3, Canada; Department of Clinical Health Psychology, Max Rady College of Medicine, Rady Faculty of Health Sciences, University of Manitoba, Winnipeg, MB R3E 3N4, Canada; Department of Psychiatry, Max Rady College of Medicine, Rady Faculty of Health Sciences, University of Manitoba, Winnipeg, MB R3E 3N4, Canada; Department of Psychology, Faculty of Arts, University of Manitoba, Winnipeg, MB R3T 2N2, Canada; Department of Community Health Sciences, Max Rady College of Medicine, Rady Faculty of Health Sciences, University of Manitoba, Winnipeg, MB R3E 0T4, Canada; Department of Psychiatry, Max Rady College of Medicine, Rady Faculty of Health Sciences, University of Manitoba, Winnipeg, MB R3E 3N4, Canada; Department of Psychology, Faculty of Arts, University of Manitoba, Winnipeg, MB R3T 2N2, Canada; Department of Community Health Sciences, Max Rady College of Medicine, Rady Faculty of Health Sciences, University of Manitoba, Winnipeg, MB R3E 0T4, Canada

## Abstract

**Introduction:**

Traumatic brain injuries are a significant concern across military populations and consequences can be long-lasting and associated with complex physical and psychological symptoms and comorbidities. For this study, our aim was to understand the prevalence and correlates of persistent post-concussive symptoms (PPCS) among active duty members and Veterans in the Canadian Armed Forces.

**Materials and Methods:**

The sample (*n* = 2,941) was from the 2018 Canadian Armed Forces and Veterans Mental Health Survey, a 16-year follow-up study of members who served in the Regular Force and were originally surveyed in the 2002 Canadian Community Health Survey—Canadian Forces Supplement. Correlates of PPCS were analyzed with unadjusted and adjusted logistic regression models.

**Results:**

Persistent post-concussive symptoms were associated with lower rank, having released from service, land uniformed environment, younger age, and shorter length of service. Individuals with PPCS were significantly more likely to have co-occurring mental disorders, chronic pain conditions, less adaptive coping styles, sleep difficulties, lower self-rated health, lower life satisfaction, and a reduced feeling of community belonging.

**Conclusions:**

Findings identified individuals with the highest odds of experiencing PPCS. Those with PPCS, compared to those without, had a higher prevalence of co-occurring symptoms and conditions, including mental disorders, chronic pain conditions, and poor psychosocial functioning. Implications include the need for interdisciplinary care to diagnose and manage complex symptoms and comorbidities, and attention to intervention and prevention efforts, involving psychoeducation, to target modifiable psychosocial factors.

## INTRODUCTION

Traumatic brain injuries (TBIs), inclusive to concussions, are the consequence of external force(s) leading to alterations in one’s brain function, activity, and/or structure(s) and have been associated with cognitive impairments, behavioral problems, physiological consequences, and psychological effects.^1-3^ Historically, recovery was assumed to follow a predictable trajectory, with most symptoms expected to resolve within days to weeks post-injury.^4^ However, symptoms can be longer lasting and chronic. Persistent post-concussive symptoms (PPCS) have been defined as TBI symptoms that continue 3-months or longer post-injury.^2^^,^^5^^,^^6^ Common persistent symptoms include headaches, memory and concentration challenges, troubles regulating mood and emotions, and daily fatigue or exhaustion.^2^^,^^7^^,^^8^ Individuals with PPCS tend to experience reduced quality of life, greater psychological dysfunction, higher risk of physical health conditions, an increased likelihood of suicidal behavior, and premature mortality.^4^^,^^7^

Military populations are particularly vulnerable to TBIs because of the high-risk nature of their occupations and occupational environments.^9^ Traumatic brain injuries in the military frequently occur in the context of warfare, most often resulting from direct combat-related impact(s) or blast-related shock waves.^1^^,^^10^^,^^11^ Reported rates of TBI in the military vary considerably across countries; in the United States, the incidence of mild TBI has been estimated to be between 15% and 23%, whereas the United Kingdom and Canada have indicated lower prevalence rates of 4% and 5%, respectively.^11^ A recent Canadian cross-sectional study further reported that nearly 10-20% of personnel returning from recent conflicts such as Operation Enduring Freedom (OEF) and Operation Iraqi Freedom (OIF) sustained a TBI, with 10-15% reporting persistent symptoms.^12^

The effect of TBIs within military populations is substantial. Compared with the general population, military personnel with TBI are more likely to spend longer periods away from work, transition out of service at higher rates, experience increased mental health challenges and suicidal behavior, and place greater demands on healthcare systems and related costs.^8^^,^^9^^,^^13-15^ Past Canadian research suggest younger personnel, males, and junior non-commissioned members are at greater risk for TBI, though little is known about which factors predict persistence of symptoms.^10^

Persistent post-concussive symptoms are frequently accompanied by mental health conditions; mood and anxiety disorders, including panic disorder (PD), are highly prevalent, and posttraumatic stress disorder (PTSD) is particularly common.^6^ Co-occurring mental health conditions may arise through several mechanisms: the traumatic nature of the TBI event itself (i.e., shared pathway); structural, cognitive, and/or metabolic changes in the brain following TBI (i.e., causal pathway); pre-morbid mental health conditions (i.e., predispositional pathway) or independent events occurring along a similar timeline (i.e., independent pathway).^2^^,^^4-6^ Evidence from U.S. Veteran populations highlight this overlap where Veterans with TBI reported significantly higher prevalence of depression (43% vs. 25%) and PTSD (31% vs. 11%) compared to those without TBI.^13^ Similarly, alcohol use disorders (AUD) are markedly more prevalent among military personnel with a history of TBI (nearly 32% vs. 10%, respectively).^16^

Chronic pain conditions are another common comorbidity, with prevalence estimates ranging from 32% to 44% among U.S. Military personnel and Veterans with TBI.^17^ Moreover, among U.S. personnel with deployment-related TBI, pain has been reported in 30-90% of cases, with headaches and back pain being among the most frequent complaints.^18^ Arthritis has similarly been disproportionately reported among deployed U.S. Service Members and Veterans with mild TBI compared to those without (43% vs. 28%, respectively).^13^ Chronic changes to gastrointestinal functions have been observed, however, prevalence data for gastrointestinal conditions in both military and civilian populations post-TBI remain limited.^19^

Given its prevalence and extensive, long-term consequences, TBI continues to be a critical focus for military research.^15^ Yet important gaps remain, particularly regarding long-term effects of TBIs in the Canadian Armed Forces (CAF). Much of the existing literature draws on U.S. military populations, often focused on Veterans actively seeking care, which may not reflect the broader experience of serving or released members. Moreover, active duty personnel–an essential group who may sustain a TBI and then redeploy–are often underrepresented in research. To date, no studies have specifically examined the prevalence or correlates of PPCS in CAF active duty or Veteran populations. Identifying and understanding the physical, psychological, and psychosocial consequences of PPCS in CAF military populations is essential for informing future assessment, intervention, and treatment strategies.

There were 3 specific aims of this study: (1) Describe individuals who endorsed PPCS among CAF members and Veterans, (2) examine the relationship between PPCS and comorbid outcomes, including mental health disorders and behaviors, and chronic pain conditions, and (3) investigate the associated effect of PPCS on an individual’s overall well-being by examining various psychosocial functioning outcomes related to general health, coping, social support, and sleep. By focusing on these objectives, we hoped to better understand a broad range of correlates of PPCS in Canadian military personnel.

## METHODS

### Participants

Participants included Regular Force personnel who had participated in the 2018 Canadian Armed Forces Members and Veterans Mental Health Follow-up Survey (CAFVMHS), a nationally representative follow-up study to the Canadian Community Health Survey on Mental Health and Wellbeing: Canadian Forces Supplement (CCHS-CFS) from 2002. The CCHS-CFS included CAF members (*n* = 5,155) ages 15-64 years old; of these members, a total of *n* = 2,941 took part in the CAFVMHS in 2018. Canadian Armed Forces Members and Veterans Mental Health Follow-up Survey included members who had since been released from the CAF (65% of sample). The total weighted response rate for the CAFVMHS follow-up survey was 68%. Respondents were interviewed by Statistics Canada trained lay-interviewers in a computer-assisted format, further details regarding methodology of the dataset can be found by Afifi et al.^20^

### Measures

#### Persistent post-concussive symptoms

Persistent post-concussive symptoms were identified using a physical health survey. The survey stated, “We are interested in ‘long-term conditions’ which are expected to last or have already lasted 6 months or more and that have been diagnosed by a health professional.” Respondents who indicated “Yes” when asked about the “effects of a TBI or concussion” on this questionnaire were classified as “PPCS,” and all others were classified as “No PPCS.”^21^

#### Sociodemographic and military characteristics

The following demographics were assessed in 2018: sex (male, female), age (continuous), marital status (partnered, unpartnered), household income ($0-$89,999, $90,000 or more), highest level of educational attainment (high school diploma or less, some post-secondary or more), military rank (Junior non-commissioned, Senior non-commissioned, Officer grouping), environmental command (air, land or sea), serving status (active duty, Veteran), ever deployed (yes, no), years of service (continuous), and number of deployments (continuous).

#### Mental health disorders

Statistics Canada trained lay interviewers assessed presence of mental health disorders using the World Health Organization Composite International Diagnostic Interview (WHO-CIDI); a reliable and valid structured interview for diagnosing mental health disorders according to the Diagnostic and Statistical Manual of Mental Disorders, Fifth Edition (DSM-5).^22^ Mental health disorders examined had a “past year” occurrence and included depression, PTSD, generalized anxiety (GAD), PD, social phobia (SP), and AUD. These were assessed individually, as a total combined “any mental health disorder” variable (yes/no), and as a “number of mental disorders” variable (continuous).

#### Suicidal behavior

Suicidal behavior was self-reported with the following 3 items: (1) had ever seriously thought about attempting suicide or taking their own life, (2) created a plan for attempting suicide, and (3) attempted suicide or tried to take their own life. Responses for all 3 items were combined into a “suicidal behavior” variable inclusive to suicidal ideation, plans, or attempts (yes/no).

#### Chronic pain conditions

Chronic pain conditions were self-reported physical health conditions that met the following criteria: (1) expected to last or already lasted 6 months or longer, (2) diagnosed by a healthcare professional, and (3) were strongly correlated with endorsement of chronic pain according to the International Classification of Diseases and Related Health Problems of the World Health Organization.^23^ Chronic pain conditions were considered current (i.e., 2018) and included arthritis, back problems, migraine headaches, and gastrointestinal conditions (irritable bowel syndrome [IBS]; inflammatory bowel diseases [IBD]; Crohn’s disease and ulcerative colitis; and intestinal and stomach ulcers). Chronic pain conditions were first individually assessed and then combined into a measure of “any chronic pain condition” (yes, no).

#### Stress and coping measures

Coping with stress was assessed with 14 items derived from the Ways of Coping Questionnaire, the COPE scale, and the Coping Strategy Indicator.^21^ Items were rated on a 4-point Likert scale, ranging from “Often” (1) to “Never” (4). Prior literature used factor analysis to group items into 3 overarching coping styles: (1) problem-focused (4 items, e.g., how often do you try and solve the problem), (2) avoidant (5 items, e.g., when stressed, how often do you avoid being with others), and (3) self-medication (3 items, e.g., how often do you use alcohol to feel better).^24^ Coping styles were dichotomized by the sample median into “low” (≤ median) and “high” (> median) endorsement.

#### Social support

Social support was self-reported using the Medical Outcomes Study (MOS) Social Support Survey; 19 items with a 5-point Likert scale, ranging from “None of the time” (1) to “All of the time” (5).^25^ The respondent’s level of social support over the past year was measured across 3 realms: affection, emotional, and informational. A combined social support variable was dichotomized at the sample median into “low” (≤ median) and “high” (> median) endorsement.

#### Sleep difficulty

Sleep difficulty was self-reported with the following item “How often do you have trouble going to sleep or staying asleep?”.^21^ Responses were measured on a 5-point scale, ranging from “None of the time” (1) to “All of the time” (5). Values of sleep difficulties were dichotomized at the sample median into those who endorsed “low” (≤median) and “high” (>median).

#### Community belonging and life satisfaction

Respondents self-rated their community belonging and overall life satisfaction. Self-ratings of community belonging responses ranged from “very strong” (1) to “very weak” (5); life satisfaction ranged from “very satisfied” (1) to “very dissatisfied” (5). Variables were dichotomized into those who endorsed a “low” (rated 1-3) and “high” (i.e., rated 4-5) level of community belonging and life satisfaction.

#### Self-reported measures of health

Respondents self-rated their overall general, physical, and mental health according to a 5-point scale ranging from “Excellent” (1) to “Poor” (5). Variables were dichotomized into those who endorsed “low” (i.e., rated 4-5) and “high” (i.e., rated 1-3) levels of general, physical, and mental health.

### Data Analysis

Stata Version 16 was used to conduct analyses.^26^ Statistics Canada provided sampling weights to account for attrition across datasets and to maintain representativeness of the original 2002 CAF target sample. To address the complex stratified sampling design, bootstrap weights were used for SE estimation. Descriptive cross-tabulations and logistic regression analyses, with 95% CI, were performed to examine prevalence and associations of sociodemographic and military characteristics, mental health disorders and behaviors, chronic pain conditions, and psychosocial correlates with PPCS. Unadjusted bivariate logistic regression models and models adjusted for sociodemographic and military variables provided unadjusted and adjusted odds ratios (i.e., ORs, AORs) to examine these relationships.

## RESULTS

The total prevalence of PPCS in the CAFVMHS sample was approximately 7%. Among those endorsing PPCS, nearly 77% were Veterans compared to active duty members.

### Sociodemographic and Military Characteristics and PPCS

Table 1 demonstrates the relationship between sociodemographic and military characteristics and PPCS. Individuals with PPCS were predominantly of male sex, partnered marital status, $90,000 or higher household income, had at least some post-secondary education, junior non-commissioned rank, Veteran (vs. active duty), land environmental uniform, had previously been deployed; with a mean age of 49.2 years, served an average of 23.5 years, and 1.5 deployments. Canadian Armed Forces personnel with PPCS were significantly more likely to be junior or senior non-commissioned members (vs. officer ranking), veterans (vs. active duty), land environmental uniform (vs. air), have a lower mean age, and lower mean years of service, compared to those without PPCS.

**Table 1.** Sociodemographic and Military Description of Sample

Sociodemographic and military characteristics	**% (95% CI)**				
	No PPCS		PPCS		OR (95% CI)
Biological sex					
Male	87.8 (87.4-88.1)		89.2 (85.7-91.9)		1.00
Female	12.2 (11.9-12.6)		10.8 (8.1-14.3)		0.87 (0.62-1.22)
Marital status					
Partnered	82.4 (80.8-83.9)		87.5 (81.0-92.0)		1.00
Unpartnered	17.6 (16.1-19.2)		12.5 (8.0-19.0)		0.67 (0.39-1.13)
Household income					
$0-$89,999	28.4 (26.5-30.3)		32.9 (25.3-41.5)		1.00
$90,000 and over	71.6 (69.7-73.5)		67.1 (58.5-74.7)		0.81 (0.55-1.19)
Education status					
High school diploma or less	42.5 (40.4-44.6)		47.1 (38.2-56.2)		1.00
At least some post-secondary	57.5 (55.4-59.6)		52.9 (43.8-61.8)		0.83 (0.57-1.21)
Rank					
Junior non-commissioned member	25.1 (23.3-26.98)		44.94 (36.8-53.4)		3.46*** (2.17-5.51)
Senior non-commissioned member	48.4 (46.5-50.3)		41.3 (33.2-49.8)		1.64* (1.02-2.64)
Officer grouping	26.5 (25.6-27.5)		13.8 (9.5-19.6)		1.00
Serving status					
Active duty	35.4 (33.4-37.3)		23.3 (16.6-31.6)		1.00
Veteran	64.6 (62.7-66.6)		76.7 (68.4-83.4)		1.80** (1.14-2.84)
Environment uniform					
Land	46.0 (43.9-48.1)		60.4 (15.5-68.6)		2.10*** (1.33-3.31)
Air	35.3 (33.4-37.3)		22.1 (15.8-30.0)		1.00
Sea	18.6 (17.0-20.3)		17.5 (12.0-24.8)		1.50 (0.86-2.62)
Deployment					
Never deployed	17.1 (15.7-18.6)		12.8 (8.4-19.2)		1.00
Deployed	82.9 (81.4-84.3)		87.2 (80.8-91.6)		1.40 (0.84-2.34)
	**Mean**	**SE**	**Mean**	**SE**	**OR (95% CI)**
Age	51.4	0.15	49.2	0.68	0.96** (0.94-0.99)
Length of service	26.2	0.15	23.5	0.77	0.96*** (0.94-0.98)
Number of deployments	1.3	0.05	1.5	0.22	1.08 (0.97-1.19)

Description of sociodemographic and military characteristics of Canadian Armed Forces personnel by PPCS status.

Bold font indicates statistical significance.

* *P* < .05,

** *P* < .01,

*** *P* < .001.

Abbreviations: CI, confidence interval; OR, odds ratio; PPCS, persistent post-concussive symptoms; SE, standard error.

### Mental Health and PPCS

Table 2 demonstrates the correlates of mental health disorders and behaviors and PPCS. Canadian Armed Forces personnel with PPCS had a significantly higher prevalence of any mental health disorder(s) compared to those without, 63.7% vs. 27%, respectively. Logistic regression models demonstrated that individuals with PPCS, compared to those without, had significantly higher odds of co-occurring depression, PTSD, GAD, SP, PD, suicidal behavior, and a higher average number of comorbid mental health disorders. After adjusting by sociodemographic and military characteristics significantly related to PPCS, identified in Table 1, associations remained significant. Overall, those with PPCS, compared to those without, were almost 4 times more likely to experience any mental health disorder (AOR) = 3.84, 95% CI, 2.66-5.55, *P *< .001).

**Table 2.** Mental Health Disorders and Comorbidities by PPCS Status in CAF Personnel

	**% (95% CI)**		OR (95% CI)	AOR^a^ (95% CI)
	No PPCS	PPCS		
Depression				
No	82.6 (80.9-84.1)	51.6 (43.2-59.9)	1.00	1.00
Yes	17.4 (15.9-19.1)	48.4 (40.1-56.8)	4.44** (3.10-6.36)	3.59** (2.46-5.24)
Posttraumatic stress disorder				
No	91.5 (90.3-92.6)	70.5 (62.0-77.7)	1.00	1.00
Yes	8.5 (7.4-9.8)	29.5 (22.3-38.0)	4.50** (2.98-6.81)	3.43** (2.15-5.45)
Generalized anxiety disorder				
No	91.4 (90.1-92.6)	72.7 (64.5-79.6)	1.00	1.00
Yes	8.6 (7.4-9.9)	27.3 (20.4-35.5)	4.01** (2.64-6.09)	3.11** (1.98-4.90)
Social phobia				
No	88.1 (86.6-89.5)	63.3 (55.1-70.8)	1.00	1.00
Yes	11.9 (0.5-13.4)	36.7 (29.2-44.9)	4.30** (2.98-6.21)	3.50** (2.36-5.19)
Panic disorder				
No	90.6 (89.2-91.9)	68.6 (89.6-76.3)	1.00	1.00
Yes	9.4 (8.1-10.9)	31.4 (23.7-40.4)	4.42** (2.84-6.87)	3.34** (2.15-5.43)
Alcohol use disorder				
No	96.3 (95.3-97.1)	95.7 (90.2-98.2)	1.00	1.00
Yes	3.7 (3.0-4.7)	4.3 (1.8-9.8)	1.15 (0.38-3.50)	1.06 (0.33-3.37)
Any mental disorder				
No	73.0 (71.1-74.9)	36.3 (28.8-44.5)	1.00	1.00
Yes	27.0 (25.1-28.9)	63.7 (55.5-71.2)	4.75** (3.33-6.78)	3.84** (2.66-5.55)
Suicidal behavior				
No	91.8 (90.5-92.9)	82.3 (74.5-88.2)	1.00	1.00
Yes	8.2 (7.1-9.5)	17.7 (11.8-25.5)	2.40** (1.44-4.00)	1.73* (1.01-2.96)
	**Mean (SE)**	**Mean (SE)**		
Number of Dx	0.57 (0.027)	1.68 (0.178)	4.33** (2.99-6.27)	3.48** (2.37-5.11)

Prevalence and Risk of Mental Health Disorders Among Canadian Armed Forces (CAF) Personnel by PPCS Status.

Bold font indicates statistical significance.

* *P* < .05,

** *P* < .001.

a Adjusted by significant 2018 descriptive variables (age, length of service, environment served, rank, and serving status).

Abbreviations: AOR, adjusted odds ratios; CAF, Canadian Armed Forces; CI, confidence interval; OR, odds ratio; PPCS, persistent post-concussive symptoms; SE, standard error.

### Chronic Pain Conditions and PPCS

Table 3 demonstrates the correlates of chronic pain conditions and PPCS. Among CAF personnel with PPCS, 85.7% reported at least one chronic pain condition, compared to 59.7% among those without PPCS. Canadian Armed Forces personnel with PPCS, compared to those without, had significantly higher odds of arthritis, back problems, migraine headaches, and gastrointestinal conditions. After adjusting by significant sociodemographic and military characteristics identified in Table 1, all associations remained significant. Overall, the likelihood of having any chronic pain condition(s) was over 3 times higher for those with PPCS compared to those without (AOR = 3.72, 95% CI, 1.25-6.16, *P *< .001).

**Table 3.** Chronic Pain Conditions and Comorbidities by PPCS Status in CAF Personnel

Chronic pain condition	% (95% CI)			
	No PPCS	PPCS	OR (95% CI)	AOR**^a^** (95% CI)
Arthritis	32.2 (30.3-34.2)	55.2 (47.0-63.1)	2.59* (1.84-3.65)	2.46* (1.73-3.50)
Back problems	43.6 (41.4-45.7)	71.6 (63.3-78.7)	3.27* (2.20-4.87)	2.95* (1.20-4.39)
Migraine headaches	9.6 (8.4-10.9)	30.9 (23.8-39.0)	4.21* (2.83-6.26)	3.70* (2.45-5.61)
Gastrointestinal conditions (IBD/IBS/ulcer/colitis)	9.4 (8.3-10.6)	25.9 (19.2-33.9)	3.37* (2.23-5.09)	2.92* (1.92-4.42)
*Any chronic pain condition(s)*	59.7 (57.6-61.7)	85.7 (78.5-90.8)	4.06* (2.42-6.80)	3.72* (1.25-6.16)

Prevalence and risk of chronic pain conditions among Canadian Armed Forces (CAF) personnel by PPCS status.

Bold font indicates statistical significance.

* *P* < .001.

a Adjusted by significant 2018 descriptive variables (age, length of service, environment served, rank, and serving status).

Abbreviations: AOR, adjusted odds ratio; CAF, Canadian Armed Forces; CI, confidence interval; IBD, irritable bowel disease; IBS, irritable bowel syndrome; OR, odds ratio; PPCS, persistent post-concussive symptoms.

### Psychosocial Factors and PPCS

Table 4 demonstrates the correlates of psychosocial factors and PPCS. Canadian Armed Forces personnel with PPCS, compared to those without, had significantly lower odds of utilizing problem-solving coping, higher odds of using avoidant coping, and higher odds of sleep difficulties. Individuals with PPCS also had higher odds of low self-rated general, physical and mental health, low life satisfaction, and low community belonging. After adjusting by significant sociodemographic and military characteristics, all previously significant associations, apart from community belonging, remained significant.

**Table 4.** Psychosocial Functioning by PPCS Status in CAF Personnel

Biopsychosocial factors	% (95% CI)		OR (95% CI)	AOR^a^ (95% CI)
	No PPCS	PPCS		
Problem-solving coping				
Low	64.4 (62.4-66.3)	75.4 (67.7-81.8)	1.00	1.00
High	35.6 (33.7-37.6)	24.6 (18.2-32.3)	0.59** (0.39-0.88)	0.64* (0.42-0.96)
Avoidant coping				
Low	59.8 (57.7-61.8)	41.0 (32.6-50.0)	1.00	1.00
High	40.2 (38.2-42.3)	59.0 (50.0-67.4)	2.14*** (1.47-3.13)	1.82** (1.23-2.68)
Self-medication coping				
Low	68.0 (66.0-69.9)	62.0 (53.1-70.1)	1.00	1.00
High	32.0 (30.1-34.0)	38.0 (29.9-46.9)	1.30 (0.89-1.90)	1.19 (0.81-1.76)
Social support				
Low	51.8 (49.6-53.9)	59.5 (51.0-67.5)	1.00	1.00
High	48.2 (46.1-50.4)	40.5 (32.5-49.0)	0.73 (0.51-1.05)	0.77 (0.53-1.13)
Sleep difficulty				
Low	70.4 (68.4-72.3)	43.7 (35.1-52.7)	1.00	1.00
High	29.6 (27.7-31.6)	56.3 (47.3-64.9)	3.07*** (2.10-4.49)	2.54*** (1.70-3.79)
General health				
High	19.0 (17.4-20.7)	45.1 (37.0-53.4)	1.00	1.00
Low	81.0 (79.3-82.6)	54.9 (46.6-63.0)	3.49*** (2.44-4.99)	2.75*** (1.88-4.01)
Physical health				
High	23.7 (22.0-25.4)	51.0 (42.6-59.3)	1.00	1.00
Low	76.3 (74.6-78.0)	49.0 (40.7-57.4)	3.36*** (2.35-4.80)	2.74*** (1.87-4.01)
Mental health				
High	20.1 (18.5-21.9)	57.2 (49.0-64.9)	1.00	1.00
Low	79.9 (78.1-81.5)	42.8 (35.1-51.0)	5.30*** (3.75-7.47)	4.24*** (2.97-6.04)
Life satisfaction				
High	7.3 (6.3-8.5)	18.8 (12.9-26.7)	1.00	1.00
Low	92.7 (91.5-93.7)	81.2 (73.3-87.1)	2.94*** (1.78-4.87)	2.25** (1.33-3.79)
Community belonging				
High	44.2 (42.2-46.3)	57.2 (47.9-65.9)	1.00	1.00
Low	55.8 (53.7-57.8)	42.8 (34.1-52.1)	1.68** (1.15-2.47)	1.47 (0.99-2.19)

Prevalence and risk of psychosocial functioning among Canadian Armed Forces (CAF) personnel by PPCS status.

Bold font indicates statistical significance.

* *P* < .05,

** *P* < .01,

*** *P* < .001.

Self-rated variables (general, physical, and mental health; life satisfaction, and community belonging) were reverse coded to show high vs. low.

a Adjusted by significant 2018 descriptive variables (age, length of service, environment served, rank, and serving status).

Abbreviations: AOR, adjusted odds ratios; CAF, Canadian Armed Forces; CI, confidence interval; OR, odds ratio; PPCS, persistent post-concussive symptoms.

## DISCUSSION

The literature surrounding TBI in military populations has grown over the past few decades, however, gaps and inconsistencies remain. To our knowledge, this is the first study to examine PPCS among CAF active duty members and Veterans and provide a comprehensive examination into a wide range of associated biopsychosocial correlates. Findings demonstrated that PPCS was significantly associated with specific sociodemographic and military characteristics, co-occurring mental health disorders and chronic pain condition(s), as well as poorer psychosocial outcomes. The findings in this study describe and elucidate the extent and correlates of PPCS among a Canadian military population.

Persistent post-concussive symptoms had a prevalence of approximately 7% among CAF members and Veterans, with a significantly higher likelihood among those who were younger, junior and senior non-commissioned members (vs. officer grouping), transitioned to Veteran status (vs. active duty), serving/served in the land environment (vs. air), and those with a shorter length of service. Our results agree with past literature finding younger age, lower rank, and serving in land/army environments to be associated with mild TBI among Canadian and U.S. Military personnel.^8^^,^^27^ Although a history of deployment is an established risk factor for TBI, our findings did not reflect this, which may be because of the majority of this sample having experienced at least one deployment or the TBI resulting outside of deployment, such as during training exercises.^9^ Together, these findings suggest that military members with PPCS may experience an earlier transition to Veteran status, coinciding with a shorter length of service and younger age.

Mental health disorders were highly prevalent among CAF members with PPCS compared to those without, with nearly 64% vs. 27% experiencing at least one, respectively. The odds of having depression, PTSD, GAD, SP, PD, and endorsing suicidal behavior were significantly higher among CAF personnel with PPCS, compared to those without. Our results agree with recent literature demonstrating a higher prevalence of mood disorders, PTSD, and suicidality among U.S. Military members^8^^,^^16^^,^^28^ and the general Canadian population^3^ post-TBI. The relationship between mental health symptoms and TBI is highly complex with overlapping symptoms contributing to lasting and comorbid outcomes. The heightened prevalence and risk for comorbid mental disorders post-TBI has been suggested to be related to numerous factors including limited social engagement and supports, lasting alterations in brain connectivity and functioning, and exposure to traumatic event(s).^6^^,^^8^^,^^15^ Mental disorders can exist before a TBI, and contribute to worsening mental health outcomes post-injury^6^; however, Brenner at al. demonstrated that new onset of mental disorders were more than twice as likely among military members post-TBI compared to those without a TBI history. This emphasizes the importance of assessing co-morbid psychological symptoms and behaviors among those with TBI post-injury. In contrast to our study, where AUD was not significantly associated with PPCS, others have found alcohol abuse or dependence to be associated with mild TBI among U.S. Military populations.^16^ Our results may have been influenced by a heightened overall use of alcohol among the Canadian military population, previously established.^29^

Chronic pain conditions were shown to have a significantly higher prevalence and increased odds in CAF personnel with PPCS. Overall, CAF members with PPCS, compared to those without, had over 4 times higher odds of experiencing any chronic pain condition. These findings agree and expand upon existing U.S. Military research that has found an increased prevalence of general chronic pain, back pain, headaches, and arthritis in personnel with TBI.^13^^,^^17^^,^^30^^,^^31^ In this study, back problems were the most prevalent among individuals with PPCS; however, migraine headaches demonstrated the strongest association with PPCS followed by gastrointestinal conditions, back problems, and arthritis. Our findings align with existing research on U.S. Veterans, where headaches and migraines were more strongly linked to TBI than other conditions associated with chronic pain.^30^ Although gastrointestinal conditions have been less studied in relation to TBI, our results were consistent with evidence that U.S. Veterans with TBI were more likely to experience a bowel disorder compared to those without TBI.^30^ The strong associations between chronic pain conditions and TBI are thought to reflect, in part, neurobiological changes in the brain primary to the injury and secondary injury mechanisms, including neuroinflammation, cellular and connectivity dysregulation, and hypersensitivity.^30^^,^^32^

The correlates of PPCS extend beyond co-occurring conditions to psychosocial outcomes. Consistent with prior studies on TBI, CAF members with PPCS demonstrated significantly lower odds of engaging in problem-solving coping and higher odds of relying on avoidant coping.^33^ In military environments, avoidant coping strategies may serve a short-term adaptive function; however, reliance on avoidance and reduced use of problem-solving strategies over time can impair one’s ability to manage everyday stressors.^34^ Engaging in maladaptive over adaptive coping strategies has elsewhere been linked to greater morbidity and prolonged disability.^35^^,^^36^

Sleep disturbances also emerged as a significant concern, with individuals with PPCS, compared to those without, reporting higher odds of sleep difficulties. This finding was consistent with past literature,^37-39^ and may reflect hypothesized shared mechanisms between secondary brain injuries post-TBI, including neuroinflammation, and sleep disruptions that can contribute to long-term and chronic sleep disorders.^40^ Given the critical role sleep plays in supporting recovery following injury, early identification of sleep difficulties and integration of sleep-focused interventions in post-TBI care may help mitigate downstream consequences, including the development and persistence of chronic pain and mental health symptoms.^13^^,^^39^

The extensive associations of PPCS were similarly observed in lower self-ratings of one’s health (general, physical, and mental), life satisfaction, and sense of community belonging seen in individuals with PPCS in this study. These findings align with, and are likely influenced by, our other findings demonstrating a high co-occurrence of mental and physical health comorbidity associated with PPCS, which may be negatively affecting an individual’s physical, psychological, and social functioning. Poor perceptions of health and well-being post-TBI have similarly been found among U.S. Veterans, who compared to those without TBI, scored significantly lower on cognitive, social, and emotional measures of psychosocial outcomes.^8^ Psychosocial functioning can be further influenced by factors such as unemployment, difficulties transitioning out of service (i.e., loss of identity, supports, peers), social isolation, relationship problems (family, romantic), and personality changes that can occur post-TBI.^6^^,^^8^^,^^15^

### Strengths and Limitations

This study has several notable strengths. It draws on a large, nationally representative dataset of CAF active duty members and Veterans, rather than a treatment-seeking clinical sample, providing a more accurate picture of the overall burden of PPCS within the CAF. Additional strengths include the use of a fully structured lay-administered diagnostic tool to assess for mental health disorders (i.e., WHO-CIDI) and the breadth of the survey, which enabled a comprehensive examination into mental health disorders, chronic pain conditions, and psychosocial factors that have been relatively underexplored in this population.

Several limitations should also be noted. First, the CAFVMHS identification of TBIs were through respondent self-report and did not include verification by clinical interview or electronic health records; therefore, TBIs and PPCS are unconfirmed and may be better understood as probable cases. In addition, the survey used 6-month criteria for meeting long-term outcomes of a TBI/concussion which is more stringent than the 3-month criteria used for PPCS in other literature; therefore, we are likely underrepresenting the scope of PPCS in the CAF. Second, analyses were unable to capture potentially critical details about the TBI/concussion, such as severity, duration, location, and the number of TBI’s sustained. Third, the dataset did not allow for examination of temporal relationships between PPCS and co-occurring conditions. As respondents were not asked about the onset of their current conditions, timing of the TBI relative to other conditions could not be established, limiting conclusions about directionality or causation. Finally, the number of individuals with PPCS limited our ability to further stratify the sample by biological sex (male, female) or serving status (active duty, Veteran), restricting the ability to identify potential differences across these groups.

### Clinical Implications

Findings indicate that assessment of mental health disorders, chronic pain conditions, and psychosocial functioning is warranted among individuals experiencing PPCS. Early identification and screening for mental health disorders, traumatic experiences, and chronic pain conditions, is key; symptom profiles may overlap and be difficult to distinguish, especially for those experiencing PPCS.^2^ The use of interdisciplinary teams of healthcare professionals could address the numerous and overlapping physical and psychological comorbidities commonly experienced by those with PPCS and provide integrated, person-centered care.

Psychosocial factors such as coping and sleep strategies may represent valuable intervention targets for reducing adverse and persistent outcomes post-TBI. Interventions that promote problem-focused coping and healthy sleep practices could help mitigate comorbidities and chronic symptoms among CAF personnel with PPCS. For example, cognitive behavior therapy (CBT) has demonstrated effectiveness in addressing related concerns such as mood and anxiety disorders and chronic pain by fostering adaptive coping strategies and highlighting the interplay between thoughts, emotions, and behaviors.^4^^,^^18^^,^^35^ Introducing CBT-informed psychoeducation and support early in CAF members’ careers may serve as an important upstream strategy, helping to reduce long-term negative outcomes during service and following transition to civilian life.

## CONCLUSION

Traumatic brain injuries can have complex and persistent consequences, contributing to biological, physical, psychological, and social functioning impairments. This study presents novel insights into numerous correlates of PPCS among CAF active-serving and Veteran members. The findings underscore important clinical implications, including the need to identify individuals at risk for PPCS, routinely screen for psychological and physical symptoms, and provide access to multidisciplinary care. Our results also highlight the importance and need for future research aimed at identifying key therapeutic targets that can support the recovery and improve outcomes for CAF members living with PPCS, and importantly, investigating ways to better avoid and reduce TBIs altogether.

## Data Availability

The original survey data for both the Canadian Community Health Survey—Canadian Forces Supplement (CCHS-CFS) in 2002, and the Canadian Armed Forces Members and Veterans Mental Health Follow-up Survey (2018) can only be accessed by application to the Canadian Research Data Centers through Statistics Canada. Statistics Canada collected and provided the data for academic and research purposes, but analyses are the sole role of the authors. The opinions expressed in this study do not represent the views of Statistics Canada.
